# Adult Sporadic Burkitt's Lymphoma Presenting with Rapid Development of Peritoneal Lymphomatosis

**DOI:** 10.1155/2017/4789706

**Published:** 2017-10-10

**Authors:** Naomi Fei, Nilay Shah

**Affiliations:** Department of Internal Medicine, West Virginia University Hospital, 1 Medical Center Dr, Morgantown, WV 26505, USA

## Abstract

Sporadic Burkitt's Lymphoma (BL) is a highly aggressive form of non-Hodgkin's lymphoma which requires prompt diagnosis and treatment. Though usual presentation involves abdominal lymphadenopathy with possible solid organ involvement, sporadic BL can rarely present with peritoneal lymphomatosis. We present a unique case with rapid evolution of BL presenting as peritoneal and omental lymphomatosis with hepatic lesions and pelvic and pericardial adenopathy.

## 1. Background

Burkitt's lymphoma (BL) is a highly aggressive subtype of B cell non-Hodgkin's lymphoma with a doubling time of 24 hours [1]. Sporadic BL is a clinical variant which comprises <1 percent of adult non-Hodgkin's lymphomas in the US [2]. Typical presentation includes abdominal symptoms of pain and distension secondary to ascites. Mesenteric and retroperitoneal lymph node enlargement is common. Extranodal involvement most commonly involves the GI tract and secondarily includes CNS, liver, spleen, kidneys, testis, and ovaries [1]. Peritoneal lymphomatosis is a rare presentation of extranodal lymphoma, usually associated with diffuse B cell lymphoma [3–5].

## 2. Case Report

An 18-year-old female, without significant medical history, presented with complaints of progressive abdominal pain and fullness. Additional symptoms included intermittent nonbloody vomiting, shortness of breath, fatigue, and low grade fevers. On physical examination, there was mild abdominal distension with tenderness to palpation in all 4 quadrants. No palpable masses were noted. No extremity edema was noted, and no cervical, axillary, or inguinal lymphadenopathy was present.

On laboratory evaluation, lactate dehydrogenase was markedly elevated (847 U/L) (nL 140–250 U/L) with uric acid (16.3 mg/dL) (nL 3–5.8 mg/dL). CA 125 level was found to be 637 U/mL (nL < 35). Additional labs were within normal limits including CBC/diff, BMP, and LFTs.

**Figure 1:**
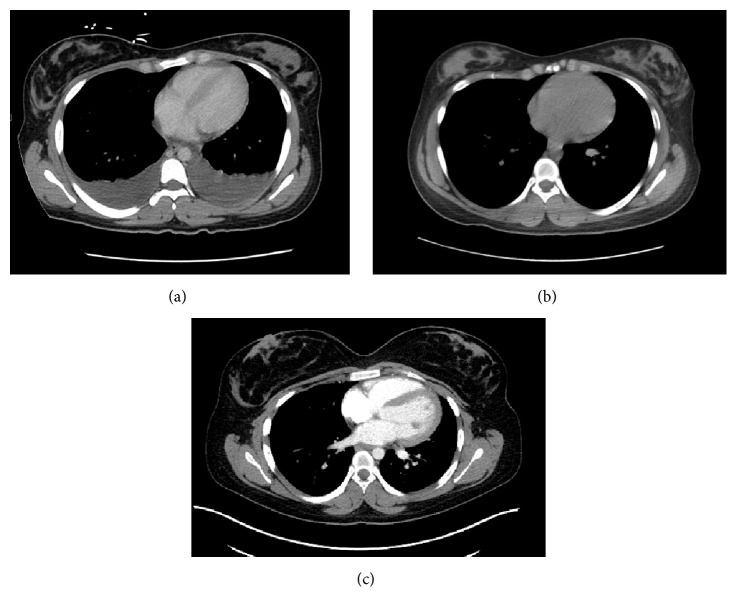
Contrast enhanced CT, axial view, upper chest. (a) Completed October 2016 with benign findings. (b) Completed November 2016 with moderate size bilateral pleural effusions. (c) Completed June 2017 with benign findings.

**Figure 2:**
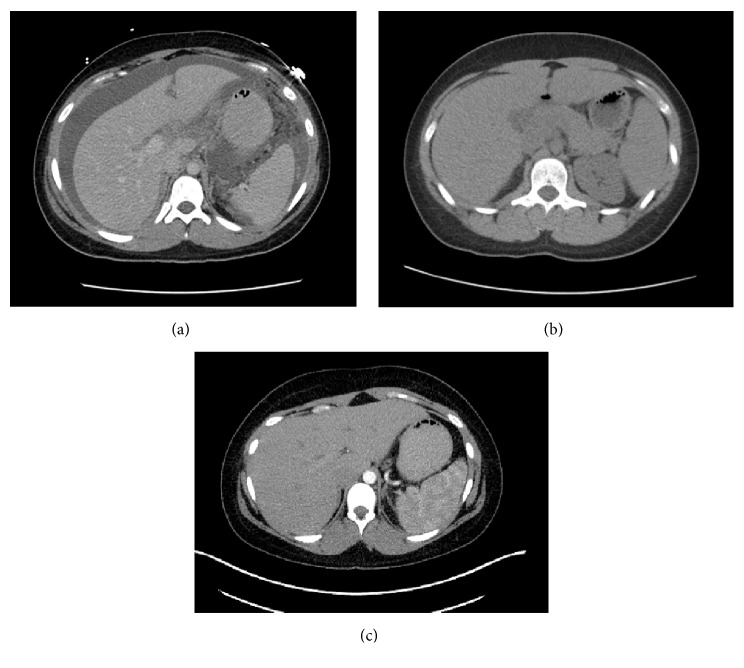
Contrast enhanced CT, axial view, upper abdomen. (a) Completed October 2016 with benign findings. (b) Completed November 2016 with multiple bilobar low attenuation indeterminate hepatic lesions. (c) Completed June 2017 with benign findings.

**Figure 3:**
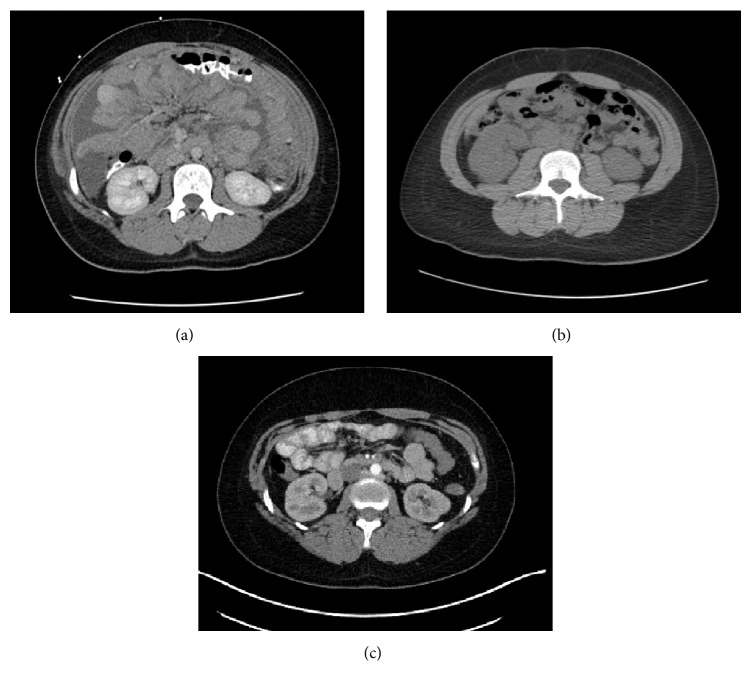
Contrast enhanced CT, axial view, mid abdomen. (a) Completed October 2016 with benign findings. (b) Completed November 2016 with extensive thickening, nodularity, and enhancement of the peritoneum and omentum. (c) Completed June 2017 with benign findings.

**Figure 4:**
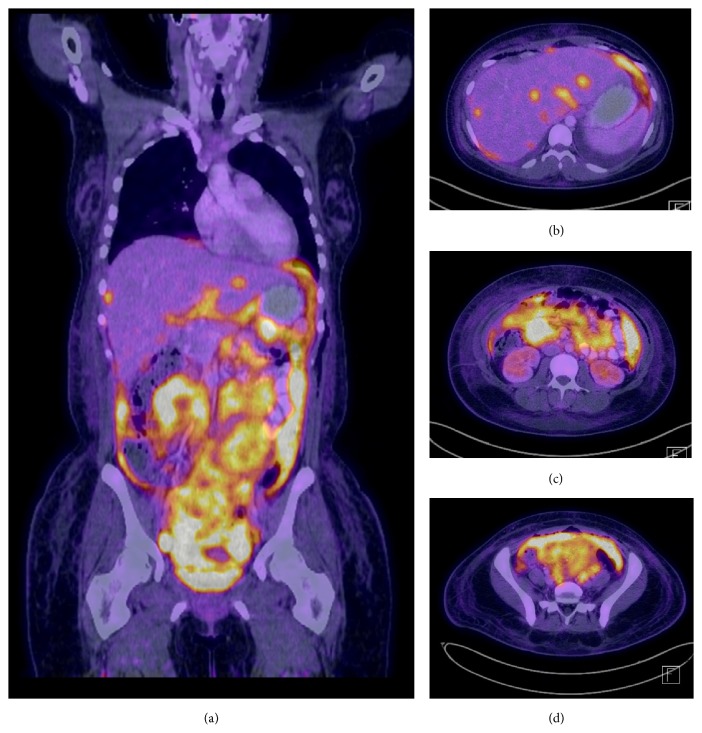
PET/CT imaging of the body performed in June 2017. (a) Coronal Body. (b) Axial upper abdomen. (c) Axial mid abdomen. (d) Axial low abdomen. Findings of diffuse and permeative abnormal hypermetabolic activity throughout the cavity corresponding to thickening and caking of the omentum and small pelvic free fluid. Hypermetabolic activity corresponding to multiple low attenuating liver lesions and retrosternal iliac chain nodes suspicious for malignancy.

**Figure 5:**
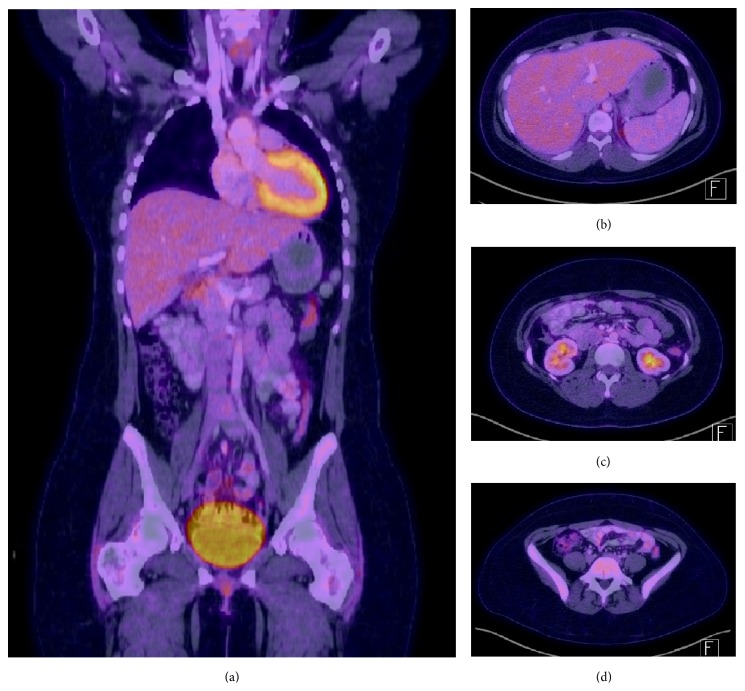
PET/CT imaging of the body performed in November 2016. (a) Coronal Body. (b) Axial upper abdomen. (c) Axial mid abdomen. (d) Axial low abdomen. Substantial resolution of patient's peritoneal involvement of malignancy and other changes suggesting malignancy noted. Previously noted liver lesions resolved.

CT abdomen and US of abdomen performed one month priorly for similar complaints were without abnormalities. On repeat imaging during admission, computed tomography (CT) of the chest, abdomen, and pelvis was significant for extensive thickening, nodularity, and enhancement of the peritoneum and omentum, read as consistent with carcinomatosis (Figures 1–3). Additional findings included multiple bilobar attenuation hepatic lesions, pelvic and pericardial adenopathy, and moderate size bilateral pleural effusions. PET/CT was significant for hypermetabolic activity throughout peritoneal cavity with omental caking (Figures 4-5). Hypermetabolic liver lesions were noted with retrosternal and bilateral iliac chain nodes also suspicious for malignancy.

Peritoneal fluid cytology was consistent with CD10+ B cell population. Biopsy of omental and peritoneal implants was consistent with Burkitt's Lymphoma. Flow cytometry identified a CD10 positive, kappa-restricted B cell population. Immunohistochemistry found CD20 positive B cells without significant expression of* BCL-2*. Ki-67 was 100%. FISH was positive for* MYC/IGH*. EBV was found to be positive. Final diagnosis was Ann Arbor stage IV Burkitt's lymphoma, with high-intermediate risk via International Prognostic Index (IPI) scoring.

Five days after presentation, the patient was started on R-Hyper-CVAD alternating with intrathecal MTX/Ara-C. Patient tolerated first 2 cycles well with follow-up PET/CT showing substantial resolution of patient's peritoneal involvement of malignancy and resolved liver lesions. Eight months after initiation of treatment, follow-up PET/CT was consistent with complete response.

## 3. Discussion

Burkitt's lymphoma (BL) is a highly aggressive subtype of B cell non-Hodgkin's lymphoma with a doubling time of 24 hours [1]. The protooncogene* c-MYC* on chromosome 8q24 is deregulated in BL allowing for disinhibition of many cell processes including cell growth, division, and death by apoptosis [6]. The World Health Organization (WHO) recognizes three clinical variants according to epidemiology, presentation, and genetic features: endemic, sporadic, and immunodeficiency associated [7].

Sporadic BL comprises <1 percent of adult non-Hodgkin's lymphomas in the US. Median age of diagnosis is 30 years. The majority of patients are male with a 3 or 4 : 1 male : female ratio. Sporadic BL is more common among Caucasians than African or Asian Americans [2].

In adults, sporadic BL typically presents with abdominal symptoms of pain and distension secondary to ascites. Mesenteric and retroperitoneal lymph node enlargement is common. Extranodal involvement commonly includes the distal ileum, stomach, cecum and/or mesentery, kidney, testis, CNS, and ovaries [1]. Peritoneal lymphomatosis is a rare presentation of extranodal lymphoma, usually associated with diffuse B cell lymphoma [3–5]. On autopsy case series of disseminated lymphoma, 64 (20%) of 322 cases had peritoneal and omental disease [8].

Peritoneal thickening is more commonly caused by peritoneal carcinomatosis secondary to spread of a primary mucinous tumor. Alternative diagnoses of peritoneal thickening include malignant peritoneal mesothelioma and tuberculous peritonitis [9]. Correct interpretation of peritoneal and omental thickening is necessary in the setting of BL given its rapid progression and nonsurgical treatment. When evaluating peritoneal thickening, radiographic signs which favor lymphomatosis rather than carcinomatosis include omental caking with peritoneal and mesenteric soft tissue nodularity and enhancement [5, 10].

It is interesting to note the elevated CA-125 level with which the patient presented. CA-125, a transmembrane glycoprotein, is derived from the epithelia originating from coelomic (pericardium, pleura, and peritoneum) and müllerian (fallopian tubal, endometrial, and endocervical) epithelia [11, 12]. Therefore, the elevation in CA-125 in this patient was likely secondary to extensive peritoneal malignancy rather than adnexal malignancy. A similar case report noting elevated CA-125 in a patient with peritoneal lymphoma has been reported by Horger et al. [13]. Inclusion of possible peritoneal sources of CA-125 elevations is crucial when developing a differential diagnosis.

This case report highlights peritoneal lymphomatosis as a rare presentation of BL with an emphasis on the rapidity of progression. Though BL often presents with diffuse abdominal involvement, to our knowledge, this is the first case report which is able to provide comparative imaging to display the rapid growth of tumor. In addition, the case report serves as a reminder of the importance of prompt histological confirmation of etiology of peritoneal thickening.

## Abbreviations

BL: Burkitt's lymphoma
WHO: World Health Organization
EBV: Epstein Barr Virus
CVAD: Cyclophosphamide, vincristine, doxorubicin, dexamethasone
MTX: Methotrexate
Ara-C: Cytarabine.
